# Stage Specific Transcriptomic Analysis and Database for Zebrafish Oogenesis

**DOI:** 10.3389/fcell.2022.826892

**Published:** 2022-06-06

**Authors:** Yoel Bogoch, Allison Jamieson-Lucy, Charles E. Vejnar, Karine Levy, Antonio J. Giraldez, Mary C. Mullins, Yaniv M. Elkouby

**Affiliations:** ^1^ Department of Developmental Biology and Cancer Research, Hebrew University of Jerusalem Faculty of Medicine, Jerusalem, Israel; ^2^ Institute for Biomedical Research, Israel-Canada, Jerusalem, Israel; ^3^ Department of Cell and Developmental Biology, University of Pennsylvania Perelman School of Medicine, Philadelphia, PA, United States; ^4^ Yale University School of Medicine, New Haven, CT, United States

**Keywords:** zebrafish (brachydanio rerio), oogenesis, transcriptomic (RNA-seq), oocyte development, meiosis

## Abstract

Oogenesis produces functional eggs and is essential for fertility, embryonic development, and reproduction. The zebrafish ovary is an excellent model to study oogenesis in vertebrates, and recent studies have identified multiple regulators in oocyte development through forward genetic screens, as well as reverse genetics by CRISPR mutagenesis. However, many developmental steps in oogenesis, in zebrafish and other species, remain poorly understood, and their underlying mechanisms are unknown. Here, we take a genomic approach to systematically uncover biological activities throughout oogenesis. We performed transcriptomic analysis on five stages of oogenesis, from the onset of oocyte differentiation through Stage III, which precedes oocyte maturation. These transcriptomes revealed thousands of differentially expressed genes across stages of oogenesis. We analyzed trends of gene expression dynamics along oogenesis, as well as their expression in pair-wise comparisons between stages. We determined their functionally enriched terms, identifying uniquely characteristic biological activities in each stage. These data identified two prominent developmental phases in oocyte differentiation and traced the accumulation of maternally deposited embryonic regulator transcripts in the developing oocyte. Our analysis provides the first molecular description for oogenesis in zebrafish, which we deposit online as a resource for the community. Further, the presence of multiple gene paralogs in zebrafish, and the exclusive curation by many bioinformatic tools of the single paralogs present in humans, challenge zebrafish genomic analyses. We offer an approach for converting zebrafish gene name nomenclature to the human nomenclature for supporting genomic analyses generally in zebrafish. Altogether, our work provides a valuable resource as a first step to uncover oogenesis mechanisms and candidate regulators and track accumulating transcripts of maternal regulators of embryonic development.

## Introduction

The production of a functional mature egg through the process of oogenesis is key for embryonic development, fertility, and reproduction. Oocyte development is a dynamic process that is tightly regulated throughout the life of an organism. In early oogenesis, this regulation orchestrates several events such as specialized mitotic divisions of oocyte precursors, transitions between cellular organization, cell polarization, and cell growth. Since these events determine the number and quality of follicles for the entire female lifespan, uncovering their underlying molecular mechanisms is crucial for a better understanding of female reproduction.

Early oogenesis processes are conserved across vertebrates, including mammals (Elkouby 2017), and the zebrafish has emerged as a versatile model for its investigation (Elkouby and Mullins 2017a). In zebrafish, oogenesis begins following the migration of the primordial germ cells (PGCs) to the genital ridge during embryogenesis and into the developing gonad in the larvae. In the gonad, PGCs give rise to germline stem cells that in turn will produce oogonial cells in a yet unclear manner. Oogonia are mitotic precursors of oocytes and undergo several rounds of incomplete cell divisions (Leu and Draper 2010). These incomplete divisions retain cytoplasmic bridges between sister oogonia, which are clustered and surrounded by somatic pre-granulosa cells, forming a conserved cellular organization of germ cells called the germline cyst (Greenbaum et al., 2007). Oogonia begin to differentiate as oocytes upon the induction of meiosis (Sánchez and Smitz 2012). Differentiating oocytes progress through the early prophase I stages, leptotene and zygotene, in the cyst and separate from the cyst, to form the primordial follicle by the pachytene stage (Elkouby et al., 2016). Oocytes in follicles are arrested at diplotene in a stage called dyctate. During this stage the primordial follicle develops into the primary follicle and continues to grow dramatically. Meiosis resumes much later during oocyte maturation.

Zebrafish oogenesis can be divided into developmental stages that are defined by oocyte size and uniquely characteristic cellular and molecular features (Selman et al., 1993). In Stage I, the primary growth stage (7–140 μm), the cells grow and progress through prophase. Considering the many distinct dynamics during specific early prophase stages, we previously provided precise staging criteria to refine Stage I into oogonia (St.Ia^*oogonia*^; 9–11 μm), and the meiotic prophase stages St.Ia^*leptotene*^ (8–9 μm), St.Ia^*zygotene*^ (10–16 μm), St.Ib^*pachytene*^ (17–19 μm), and St.Ib^*diplotene*^ (20–140 μm) (Elkouby and Mullins 2017a; Elkouby 2017). In this nomenclature, “Ia” indicates oocytes in the cyst, and “Ib” indicates oocytes in the follicle, as previously determined (Selman et al., 1993).

In parallel to these early meiotic events, a large RNA-protein (RNP) granule, called the Balbiani body (Bb), forms and is a prominent feature in oocytes from insects to humans (Elkouby and Mullins 2017b). In zebrafish and most vertebrates, the Bb establishes oocyte polarity along the animal-vegetal axis, which is key for embryonic development (Kloc et al., 2004; Bontems et al., 2009; Gupta et al., 2010), and in mammals it was suggested to contribute to the development of the primordial follicle (Marlow and Mullins 2008). In zebrafish, Bb components polarize around the centrosome at St. Ia^*zygotene*^ and continue to nucleate within an indentation in the nuclear envelope termed the nuclear cleft, during St. Ib^*pachytene*^ to early St. Ib^*diplotene*^ (Elkouby et al., 2016). The mature Bb is completely formed while the nuclear cleft rounds out by mid-St. Ib^*diplotene*^. The Bb forms around the centrosome and is present in equivalent stages of oogenesis also in insects and mice (Lei and Spradling 2013; Tworzydlo et al., 2016; Elkouby 2017). The Bb ultimately dissociates at the oocyte cortex at the end of St. Ib^*diplotene*^ (Gupta et al., 2010; Escobar-Aguirre et al., 2017a).

In Stage II, the cortical alveolus stage (140–340 μm), the oocyte begins to produce cortical granules (also called cortical alveoli) which will later be released at fertilization. In addition, the vitelline envelope begins to form, and by the end of the stage the envelope reaches its maximum thickness. In Stage III, the vitellogenesis stage (340–690 μm), the yolk develops and consequently the oocyte increases vastly in size. During Stage IV, oocyte maturation (690–730 μm), meiosis resumes until arresting at the second meiotic metaphase. Finally, the mature egg, Stage IV, is ovulated (Selman et al., 1993).

Many regulatory genes that are expressed during these stages and control these processes have been recently identified in zebrafish. These include for example, the oocyte specific transcription factor Figla (Qin et al., 2018), the piRNA pathway proteins ziwil1 and ziwil2 (Houwing et al., 2007; Houwing et al., 2008), ligands like Wnt4 (Kossack and Draper 2019; Kossack et al., 2019), Tdrd12 (Dai et al., 2017), meiotic regulators Rad21l1 (Blokhina et al., 2021) and spo11 (Blokhina et al., 2019), Bb regulators buckyball, macf1 and tdrd6a (Marlow and Mullins 2008; Escobar-Aguirre et al., 2017a; Roovers et al., 2018), and growth hormone 1 (Gh1), fshr, and the androgen receptor (Chu et al., 2015; Zhang et al., 2015; Crowder et al., 2018; Yu et al., 2018). The precise molecular mechanisms underlying many of these processes are still unclear. Systematic transcriptomic analysis of oogenesis has great potential to identify stage specific candidate regulators and markers. However, to our knowledge, a transcriptomic analysis that can systematically identify stage specific genes in all early stages of oocyte development in zebrafish has not been performed.

Here, we performed RNA sequencing of five oocyte stages encompassing stages I to III. We identified and compared thousands of differentially expressed genes across these stages of oogenesis and determined gene ontology for specific stages. These data provide the first stage specific molecular description for oogenesis in zebrafish, identifies two prominent developmental phases in oocyte differentiation, and shows the expression of maternal embryonic regulators as they accumulate in oocytes. This stage specific oocyte transcriptomic data, which we deposited online in NCBI Sequence Read Archive (SRA https://www.ncbi.nlm.nih.gov/sra/?term=SRP360172, and https://www.ncbi.nlm.nih.gov/sra/?term=SRP360207) as a resource for the community, lays the foundation for identifying proteins and signaling pathways as potential novel regulators of zebrafish oogenesis.

## Methods

### Oocyte Isolation

Ovaries were dissected from wild type Tü females at reproductive age (8–12 months post-fertilization), and oocytes were isolated according to Elkouby and Mullins 2017 (Elkouby and Mullins 2017a). Briefly, ovaries were digested in a mix of Collagenase I, Collagenase II, and Hyaluronidase for 10 min at RT. The supernatant containing the oocytes was passed through a cell strainer of the upper size limit of the group of interest. The cells were then passed through a sieve with the lower size limit and the smaller cells were discarded. The cells were washed with HL-15 and collected by centrifugation. Cells were flash-frozen for RNA extraction. All groups were processed in biological duplicates. For each oocyte group and duplicate, ovaries from two female fish were pooled together.

Ovaries that were isolated for imaging were derived from same aged females of Tg(ub:zebrabow) (Pan et al., 2013), or double transgenic Tg(ub:zebrabow);Tg(vasa:GFP) fish.

### RNA-Sequencing

After RNA extraction, samples were treated with oligo (dT) beads to enrich for poly(A)+ RNA, according to the manufacturer protocol. RNA-seq libraries were prepared using strand-specific TruSeq Illumina adapters and sequenced by the Yale Center for Genome Analysis. For record keeping and bioinformatics analysis, samples annotations were stored in LabxDB (Vejnar and Giraldez 2020). The “export_sra” and “export_sra_fastq” tools from LabxDB (Vejnar and Giraldez 2020) were employed to export sequencing data to SRA. Raw reads for this study are publicly accessible in the Sequence Read Archive under project SRP360207and SRP360172.

### Bioinformatic Analysis

Reads were processed with Cutadapt, v1.12, to remove low quality and adapter sequences, then filtered with fastq_quality_filter of the FASTX package, v0.0.14, to remove overall low quality reads. Processed unique reads were aligned to the zebrafish genome, GRCz11, with TopHat, v2.1.1, using Ensembl gene annotations from release 92 and allowing for up to 5 mismatches per read. Htseq-count, v0.6.0, was used to obtain raw counts, which were analyzed with the R package DESeq2, v1.12.4, for normalization and differential expression. Genes with a sum of raw counts less than 6 over all samples were filtered out prior to normalization.

Differential expression was tested with two different statistical models: 1. A likelihood ratio test (LRT), comparing the division to the different stages (full model) and the intercept (reduced model). 2. Pairwise comparisons between each pair of stages, using the default Wald test. In both analyses, default parameters were used, except setting the significance threshold as padj <0.05 and setting independent Filtering to false. Genes were used for further analysis if they had minimal expression in the system, baseMean>5, and their fold change was both significant and passed a baseMean-dependent threshold, requiring higher fold for lowly expressed genes and a milder fold for highly expressed genes. The exact formula for this baseMean-dependent cutoff was |log2FoldChange| > 5/baseMean^0.5 + 0.6. Data was visualized with R, v3.3.3, using packages “RColorBrewer_1.1–2,” “pheatmap_1.0.8,” “ggplot2_2.2.1” and “ggrepel_0.7.0”.

### Zebrafish-Human Orthology

Molecular function bioinformatic analysis of the significantly differentially expressed genes (DEGs) (Padj <0.1) of each RNASeq comparison: Nuc vs. Symbrk (IN/IS), MatBb vs. Nuc (IM/IN), Stage II vs. Nuc (II/IN), and Stage II vs. MatBb (II/IM) was carried out using the Ingenuity Pathway Analysis (IPA®) (QIAGEN Inc. https://digitalinsights.qiagen.com/products-overview/discovery-insights-portfolio/content-exploration-and-databases/qiagen-ipa/).

As input for IPA® we used the human orthologs of the zebrafish genes. Based on data curated in ZFin and *ensemble* 15,122 genes (60%) of the 25,298 protein coding genes in zebrafish had annotated human orthologs. In the remaining 10,176 genes (40%) human orthologs can be only indicated as associated. When orthology is assigned from within IPA®, it considers only the zebrafish genes with 1:1 orthology to human (i.e., a human gene with a single zebrafish ortholog), which are 64.3% of the cases (9722/15122). However, for 35.4% of the zebrafish genes with human orthologs (5361/15122), there is a one-to-many orthology, i.e., one human gene is an ortholog to more than one zebrafish genes, typically two. To avoid losing the differential expression information from these genes, we added a directionality check, described below.

We first obtained the orthology assignment from the manually curated zebrafish information network (ZFIN, April 2019). In cases of 1:1 orthology we simply used the human ortholog. In cases of one-to-many orthology, we applied the directionality test: cases where all the zebrafish DEGs with the same human ortholog changed their expression in the same direction were included in the analysis; cases of opposite significant change were omitted from the DEGs lists and the analysis. In about 80% of the cases (the 35.4% with many orthologs) where two DEGs had the same human ortholog, their expression changed in the same direction. Altogether, this approach has enabled us to get a much larger coverage of orthologs (92.6% of the 60% of total zebrafish genes that have annotated human orthologs) and hence more informative experimental data. The remaining 7.4% requires individual manual investigation. A limit in this approach is the lack of analysis of the 40% of genes with no curated orthologs.

### Bioinformatic Functional Analysis Tests

For functional analysis of the clusters we used the Ingenuity Pathway Analysis (IPA®) (Qiagen, https://digitalinsights.qiagen.com/products-overview/discovery-insights-portfolio/content-exploration-and-databases/qiagen-ipa/). For the pairwise analysis we used the WebGestalt (Liao et al., 2019) gene ontology database with a cutoff of FDR<0.05.

### Immunoflorescence and Microscopy

Whole ovaries from Tg(ub:zebrabow);Tg(vasa:GFP) double transgenic line were dissected, fixed, and immunofluorescence labeling was performed as in Elkouby and Mullins (Elkouby and Mullins 2017a), with anti-GFP antibody (A11122, Invitrogen) to label transgenic GFP. Images were acquired on a Zeiss LSM 880 confocal microscope using a 40X lens.

For imaging isolated oocytes, oocytes were placed in a 24-well dish following isolation, and images were acquired on a Nikon TL microscope, equipped with an incubation chamber set for 28°C, and using a 10X lens. Acquired images were not processed, and only contrast/brightness were slightly adjusted.

## Results

### A Stage Specific Transcriptomic Analysis of Oogenesis Reveals Highly Dynamic Gene Expression Throughout Oocyte Development

A molecular characterization of different stages in oogenesis requires separation of oocytes according to the developmental stages of interest. Based on the characteristic size range that defines each stage (Elkouby and Mullins 2017a), we isolated oocytes of different developmental stages by size. However, the resolution of cell size separation did not enable us to exclusively separate individual sub-stages in Stage I. We focused on groups of oocyte stages based on our updated staging criteria of St. I, combined with key events in Bb formation. We reasoned that these ranges of specific stages represent distinct steps in oocyte developmental biology. Oocytes were separated according to Elkouby and Mullins (Elkouby and Mullins 2017a), briefly:

1) Symmetry breaking (termed Symbrk)—size 8–20 μm. This size includes St. Ia^*oogonia*^, St. Ia^*leptotene*^, St. Ia^*zygotene*^, and St. Ib^*pachytene*^. This group represents mitotic oogonia, as well as oocytes at the onset of meiosis and early prophase, including events like oocyte symmetry breaking, and formation of the chromosomal bouquet.
2) Nuclear cleft (termed Nuc)– size 15–50 μm. This size includes St. Ib^*pachytene*^ to mid St. Ib^*diplotene*^.
Oocytes in this group undergo nuclear cleft formation, Bb maturation, and the beginning of folliculogenesis.
3) Mature Balbiani body (termed MatBb)—size 35–100 μm. This size includes later St. Ib^*diplotene*^, representing oocytes in primary follicles with mature Bb.
4) Stage II—Size 100–300 µm. This size includes late St. Ib^*diplotene*^ and mostly Stage II. In oocytes in this stage, vegetal and animal RNAs localize to their corresponding cortex poles, and cortical granules form.
5) Stage III—Size >300 µm. This size includes Stage III and Stage IV, however, we selected against Stage IV by manually selecting opaque cells (Stage IV oocytes are transparent), enriching Stage III, which are undergoing vitellogenesis.

**FIGURE 1:**
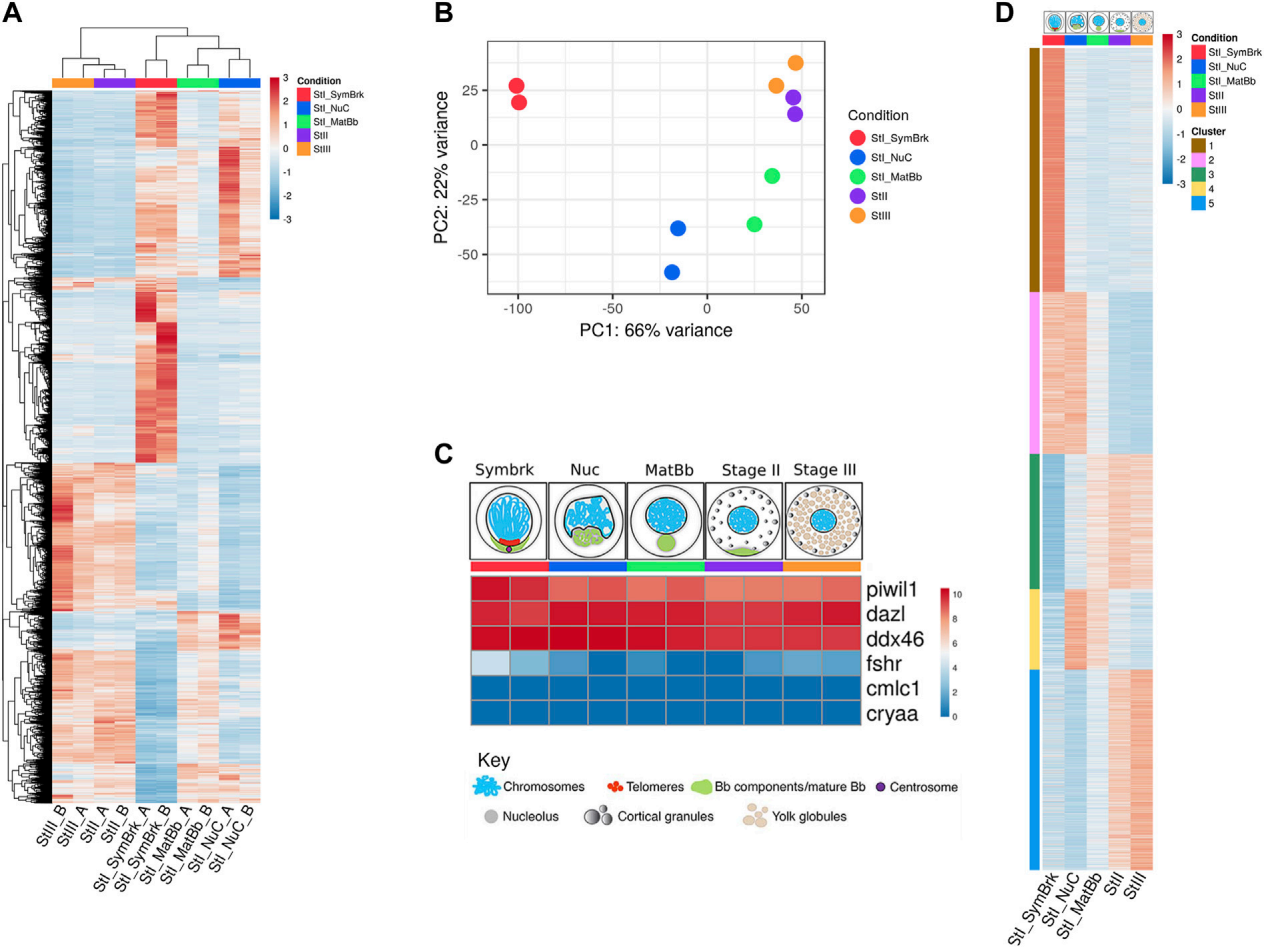
A high throughput analysis of 5 stages of oocyte development. **(A)** Unsupervised hierarchical clustering of all genes that were changed between any of the stages. Samples are ordered according to clustering. **(B)** PCA of the duplicates of the different stages. **(C)** Control gene expression confirms the accuracy of our data. Germ cell specific genes are expressed, but somatic markers show background expression comparable with expression of non-ovarian genes images of oocyte stages groups were modified from Elkouby and Mullins (Elkouby and Mullins 2017a). **(D)** Heat map of k-means clustering of genes that were differentially expressed as determined by a likelihood ratio test. Five clusters were determined to allow visualization of the expression trends. Each stage is represented by a color throughout the figures: red for Symbrk, blue for Nuc, green for MatBb, purple for Stage II, and orange for Stage III. Red denotes higher expressed genes and blue denotes lower expressed genes. The numbers are normalized relative expression. Each column is the average of the duplicates.

Each group was sequenced in duplicate. Strikingly, 11,011 genes were differentially expressed throughout the five groups of developing oocyte stages (Figure 1A). Principal component analysis (PCA) showed that each duplicate clustered together and that the closest stages in terms of gene expression are Stage II and Stage III (Figure 1B), which is also seen in the clustering in Figure 1A. Germ cell marker genes, such as piwi, dazl and ddx4 (*vasa*) (Lasko and Ashburner 1988; Wagner et al., 2004; Houwing et al., 2007) were highly expressed across all stages as expected (Figure 1C). Expression of a somatic follicle cell gene (fshr) was close to undetectable levels and comparable to non-ovarian gene expression like heart (cmlc2) and eye (cryaa) markers, throughout the Nuc, MatBb, StageII and Stage III groups (Figure 1C). Expression of these marker genes confirm that these groups specifically represent oocytes.

The Symbrk group was distinctly separate from the other groups as shown by PCA (Figure 1B). Since this group included very small cells (<20 μm) we assume that in addition to germ cells, it may contain various somatic cell types, including granulosa, as well as other ovarian cells like endothelial cells and tissue resident immune cells. Indeed, fshr expression was slightly higher specifically in this group (Figure 1C). To test for the presence of somatic cells in the groups of small oocytes, we imaged SymBrk and Nuc isolated oocytes. We first empirically found that Tg(ub:zebrabow) transgenic fish express the zebrabow-encoded RFP specifically in somatic cells in ovaries, and not in germ cells. We generated a double-transgenic line Tg(ub:zebrabow);Tg(vasa:GFP), encoding zebrabow-RFP and GFP driven by the promoter of the germ cell specific marker *vasa*. These double-transgenic ovaries confirmed the exclusion of zebrabow-RFP from GFP-positive germ cells, and its expression only in somatic cells (Supplementary Figure S1A). We therefore used zebrabow-RFP as a reliable ovarian somatic-cell marker, and imaged isolated oocytes from Tg(ub:zebrabow) ovaries.

As expected, while most cells in the SymBrk group were negative for RFP, this group included cells that were positive for RFP, indicating the presence of somatic cells (Supplementary Figure S1B top), and that transcripts from this group likely represent a mixture of oocytes and somatic cells. In contrast, Nuc samples isolated from Tg(ub:zebrabow) ovaries were negative for RFP. We only detected ∼2% RFP-positive cells in this group (Supplementary Figure S1B bottom), concluding that gene expression from this group reliably represents the oocyte transcriptome specifically. As discussed below, we removed the SymBrk group from our following comparative analyses. We conclude that we successfully captured the unique transcriptomic signature of each stage range in oogenesis, at least for the Nuc, MatBb, Stage II, and Stage III groups.

To determine trends of gene expression that could represent developmental biological processes in oogenesis, we performed k means clustering of genes that were ascertained as differentially expressed between any group by the likelihood ratio test (see Methods). Using unbiased clustering resulted in 12 clusters (Supplementary Figure S2), exhibiting finely detailed changes in gene expression. Several clusters included genes expressed in pairs of stages, for example clusters #1 included genes expressed in Symbrk and Nuc, and cluster #2 those in Stage II and Stage III. We also found that many of the clusters were similar, distinguishing only mild changes in gene expression trends. Therefore, to first focus on major trends of transcriptomic changes, we narrowed our analysis to 5 representative clusters (Figure 1D). Cluster #1 includes genes that were exclusively upregulated in Symbrk. Cluster #2 includes genes that were upregulated in both Symbrk and Nuc and downregulated in Stage II and III. Cluster #3 included genes that had increasing expression across stages with the lowest expression in Symbrk and highest in Stage III. Cluster #4 included genes with higher expression specifically in the Nuc and MatBb stages, while cluster #5 had higher expression only in Stage II and Stage III. We hypothesized that these five trends of gene expression can molecularly describe biological changes throughout oocyte development, and we next focused on their analysis.

### Converting Zebrafish Gene Names to Human Nomenclature for Bioinformatic Analyses

To gain insight into the indicated cellular functions from our dataset, we wanted to perform a functional enrichment analysis on differentially expressed genes from the different clusters. However, a straightforward analysis was challenging due to technical issues. First, the zebrafish is only partially annotated by Gene Ontology (GO), which relies mainly on mammalian literature. Second, a genome duplication event in the teleost lineage resulted in more than one gene paralogs for many genes in zebrafish (Postlethwait et al., 1998; Howe et al., 2013), complicating GO analysis that is based on curated data for single mammalian genes. Finally, based on curated data on ZFIN and ensemble, 60% (15,122 genes) of the total zebrafish coding genes have annotated human orthologs. We therefore converted gene names from zebrafish to human nomenclature for 60% of the zebrafish coding genome, which has human orthologs (Supplementary Data sheet S1; Methods).

From these 60%, 64.3% of zebrafish genes had a single human ortholog. In 35.4% of zebrafish genes, typically two zebrafish genes had a single human ortholog. Using a directionality test (Methods) resulted in conversion of ∼80% of these (35.4% with two zebrafish paralogs) genes to their human counterparts for IPA and GO analyses. A limit of this conversion is the variability between gene paralogs that can function redundantly or in specialized manners in distinct developmental and cellular contexts (for examples see (Meyer and Schartl 1999; Oltova et al., 2018; Bao et al., 2019)), which might skew interpretations, and these were the 20% of the 35.4% that did not pass our directionality test. In addition, 40% of all zebrafish coding genes exhibit some human orthology association, but we did not include these uncertain associations in our analysis. However, in determining ontology and functional terms, GO combines groups of genes with similar expression dynamics, and not individual genes. Therefore, the use of this conversion in this analysis is very likely to provide significant insight into cellular functions, as a first step towards more specific functional investigations. Our zebrafish-human gene nomenclature converting table together with the directionality test approach enabled broad GO analyses of close to 60% of the zebrafish coding genome in our analysis. This approach can be used in gene expression and functional term analysis beyond oogenesis in any investigative context in zebrafish, and we are providing the list of zebrafish-human ortholog assignment as a resource.

### Five Clusters of Gene Expression Dynamics Molecularly Describe Oocyte Differentiation

Using our zebrafish-human gene nomenclature conversion, we performed functional enrichment analysis on differentially expressed genes from the five clusters we identified above. This analysis revealed functionally enriched terms and processes that were unique to each cluster, as described next.

**FIGURE 2:**
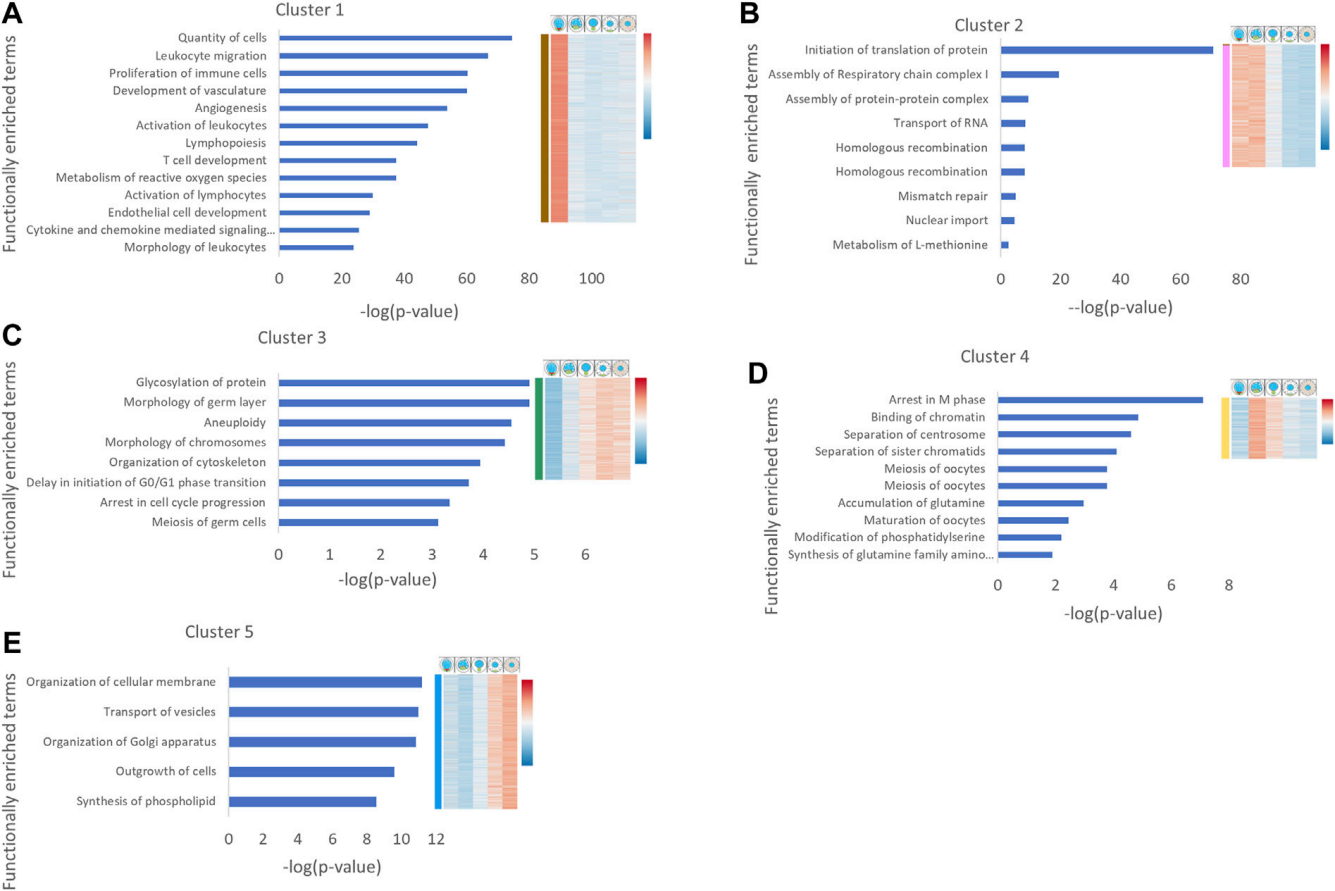
Functional enrichment analysis of gene expression clusters in oogenesis. Human homologues of genes from each cluster were analyzed by IPA. Cancer related functions were removed. The main functionally enriched terms for each cluster are shown. The *X* axis is the [–log (*p*-value)] corrected for FDR. Heat map on the right shows the corresponding cluster expression pattern derived from Figure 1C. Cartoons above the heatmap depict the different stages. The heat map shows relative expression of genes, where red denotes higher expressed genes and blue denotes lower expressed genes.

The main functional terms enriched in cluster #1 were associated with the immune and vascular systems, likely representing ovarian resident immune cells and blood vessel cells that were captured in this group (Figure 2A), as we suspected above (Figures 1B,C). This is consistent with our observation above that the SymBrk group includes somatic cells, and therefore this cluster includes genes from somatic ovarian cells. Since this sample likely contains transcripts from multiple cell types that are indistinguishable in RNA sequencing in bulk, we decided to remove this group from further analyses, and instead, use cluster #2 that better represents germ cells at these early stages (see below). Interestingly, this enrichment in cluster #1 indicates the significant presence of resident immune cells in the ovary, which to our knowledge has not been previously addressed.

Cluster #2 includes genes that are specifically enriched in Symbrk and Nuc stages. Functionally enriched terms in this cluster included meiotic processes such as homologous recombination and mismatch repair (Figure 2B), consistent with the included leptotene-pachytene prophase oocytes in this sample, when DNA double-strand breaks are repaired through homologous recombination, as well as nonhomologous repair mechanisms (Imai et al., 2021). Interestingly, enriched terms also included RNA transport and translation (Figure 2B), which are consistent with oocyte symmetry breaking and polarization of localized mRNA through Bb formation at these stages. Another interesting term was “Assembly of respiratory chain complex I,” which is part of the mitochondrial electron chain reaction respiratory complex (Signes and Fernandez-Vizarra 2018). The Bb was suggested to select for and aggregate the most fit and active mitochondria in the oocyte (Cox and Spradling 2003; Bilinski et al., 2017), and it is plausible that mitochondria undergo modifications during Bb formation. Considering the confirmed specificity of the Nuc group (Figure 1C) and the enrichment of the specific meiotic functional terms in this cluster (Figure 2B), we conclude that instead of cluster #1, cluster #2 better represents germ cell transcripts at these early stages of oogenesis, and that we were able to identify these in our analysis.

Cluster #3 contains genes with gradually increasing expression through the stages. Functionally enriched terms in cluster #3 included several meiosis functions, consistently with the progression of the meiotic cell cycle from prophase to dyctate, and then preparation for nuclear envelop breakdown towards oocyte maturation (Elkouby and Mullins 2017a) (Figure 2C). The enrichment of the term “Organization of cytoskeleton” (Figure 2C) is consistent with oocyte growth, and the term “Glycosylation of protein” (Figure 2C) is congruent with the required glycosylation of zona pelucida proteins. Interestingly, the term “Morphology of germ layers” was also enriched in this cluster (Figure 2C), which likely contains transcripts of maternal regulators of embryonic development. Indeed, genes such as ctcf (Wan et al., 2008), eIf4g (Keiper 2019), alk4 (Souquet et al., 2012), and *hira* (Nashun et al., 2015; Burkhart et al., 2020) have all been shown to play essential roles in oocyte development and are maternally deposited. All other genes associated with this functional term are maternally deposited according to ZFIN expression data and include components of signaling pathways that pattern the early embryo such as Nodal and Wnt/β-cat (Whitman 2001; Souquet et al., 2012; Fuentes et al., 2020; Habara et al., 2021) (Supplementary Data sheet S2).

Formation of germ layers and patterning of the early embryonic axes heavily relies on maternally deposited transcripts of regulators that begin to act prior to zygotic genome activation (Escobar-Aguirre et al., 2017b). When exactly these are transcribed in oogenesis, and whether they are expressed synchronously and/or by common regulation is unknown. Our analysis shows that maternal embryonic regulators begin their expression as early as St. Ib^*diplotene*^ and increase over time. Further investigating the genes in this group and generally in our dataset, could be used to determine whether genes of interest are maternally deposited, which is an important consideration for their functional analysis.

Cluster #4 includes genes that are upregulated in the Nuc and MatBb stages. Functionally enriched terms in this cluster showed progression through meiosis, which is consistent with progression from pachytene to diplotene in oocytes included in this group. Similarly, identified enriched terms of oocyte development are consistent with the formation of the primordial follicle and development to the primary follicle at these stages (Selman et al., 1993). Interestingly, metabolic functions, including glutamine synthesis and phosphatidylserine modifications were identified, indicating that these metabolic pathways are specifically associated with the primary growth St. I in oogenesis, where the oocyte grows from 15 to 100 μm (Figure 2D).

The similar but distinct terms of membrane modification “organization of cellular membrane” and “synthesis of phospholipid” are enriched in cluster #5, which includes genes upregulated in stages II and III (Figure 2E) that likely correspond at least in part to the massive growth in lipid encased yolk globules and cortical granule vesicles that form during these stages. This indicates a continuous and likely developmentally regulated growth and modifications of the cytoplasmic and organelle membranes as the oocyte grows to over 300 μm in diameter and *r* ^3^-fold in volume at St. III (Selman et al., 1993). Additionally enriched terms here were “outgrowth of cells” and “transport of vesicles” (Figure 2E), which further point to oocyte growth as a prominent process at these stages.

Altogether, the functionally enriched analysis has confirmed stage appropriate oocyte functions, while uncovering intriguing unique functions in each stage. The general theme arising is a shift from meiotic processes and RNA regulation early in oocyte differentiation to metabolic functions and cellular growth processes later, as the oocyte massively grows. In parallel, the oocyte seems to gradually accumulate transcripts of maternal embryonic regulators towards future maturation and fertilization.

**FIGURE 3:**
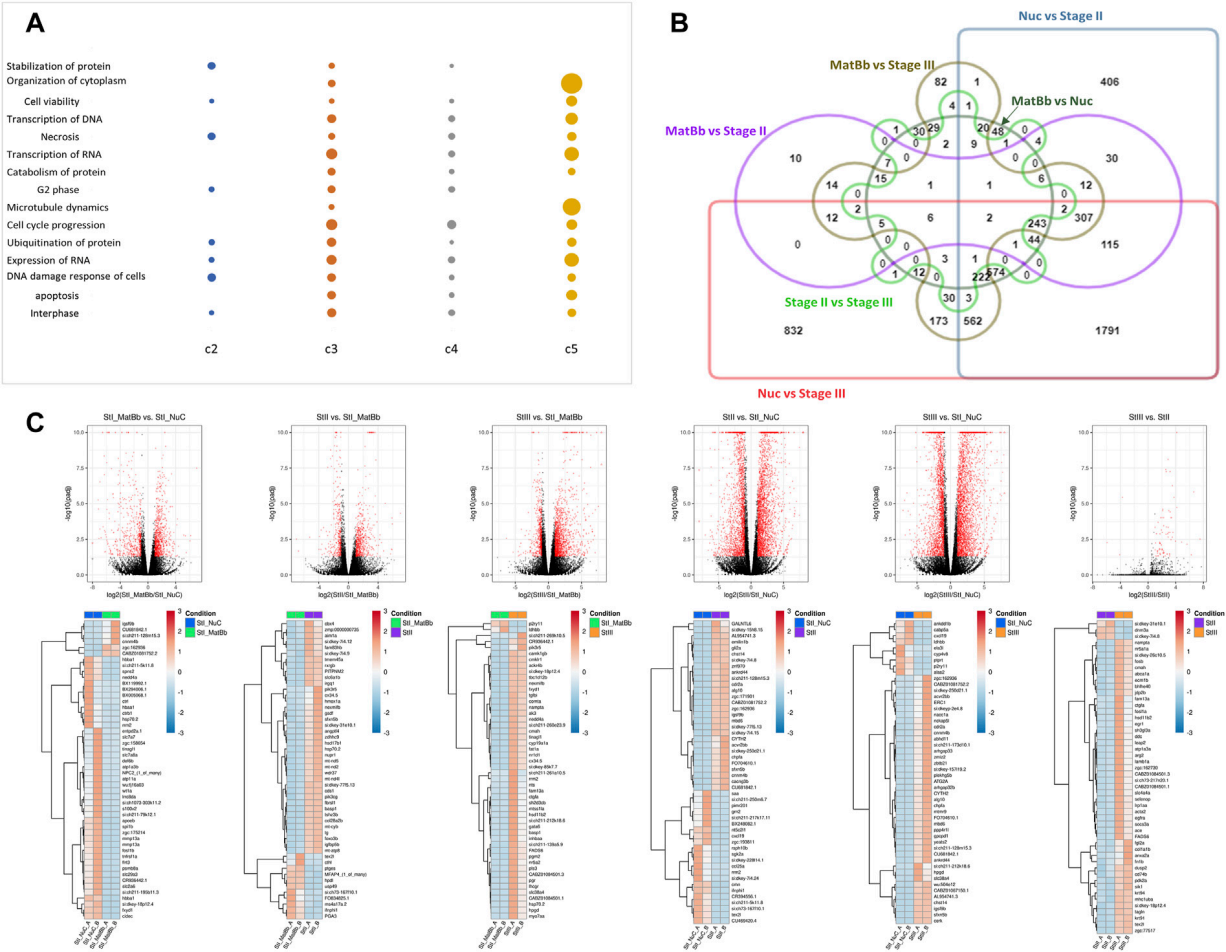
Analysis of differentially expressed genes of different stages. **(A)** A dot plot of functionally enriched terms that were associated with at least 3 different clusters. The size of the dot is representative of the [– log(*p*-value)] corrected for FDR. The larger the dot the smaller the *p* value. A missing dot means that the specific function was not found in the cluster. **(B)** Edwards venn diagram of differential expression of genes between all pairs. The Symbreak stage was omitted from the analysis. Each pairwise analysis is denoted by name, color, and shape. Genes were determined as differentially expressed if they had a fold change of at least 2 and FDR<0.05. **(C)** Top: Volcano plots of pairwise analysis of DEGs. Red dots are for significant genes, black dots are for all other genes (not significant). Bottom: Heat maps for the top 50 significantly expressed genes according to absolute fold change.

Several processes in this analysis were similar for at least 3 clusters (albeit with different genes). Among the most common general functions are DNA damage response, cell cycle progression, microtubule dynamics, organization of cytoplasm, and others (Figure 3A). Although these functions are quite broad, they are relevant to the developing oocyte. Furthermore, there are some differences in the level of expression of these functions, for example organization of cytoplasm seems to be associated strongly with Stage II and III compared to the others.

### Pairwise Comparisons Between Stages

We next investigated the differentially expressed genes (DEGs) between specific pairs of oocyte developmental stages. As mentioned above, the Symbrk group contained transcripts of somatic ovarian cells, and we therefore excluded it from the pairwise analysis. Comparisons of the DEGs from all pairwise comparisons showed that there are hundreds of genes uniquely expressed between Nuc and Stage III and between Nuc and Stage II while the rest of the comparisons had tens of unique DEGs. (Figure 3B). Comparing the Nuc stage to Stage II or Stage III revealed 412 and 833 genes, respectively, which are differentially expressed specifically between these stages. This large number of DEGs between these stages reveals a major leap in gene expression at this point in oogenesis (Figure 3B). Such a leap in gene expression changes is concomitant with 1) the shift from early meiotic and RNA regulation events to later metabolic and cellular growth processes, and 2) the accumulation of expression of maternally deposited genes, both of which we detected in our cluster analysis.

Many DEGs that were identified as unique to each pairwise comparison, are of scientific interest and can be used to differentiate between two specific stages. Figure 3C shows the volcano plots of all pairwise comparisons and their corresponding top 50 significant DEGs based on absolute fold change (see Supplementary Figure S3 for larger view images of the heat maps and gene names). Heat maps of top 50 DEGS in each comparison based on significance (*p*-value corrected for FDR) are shown in Supplementary Figure S4. Interestingly almost no DEGs were identified between Stage II and Stage III reflecting the similarity seen in the cluster analysis.

**FIGURE 4:**
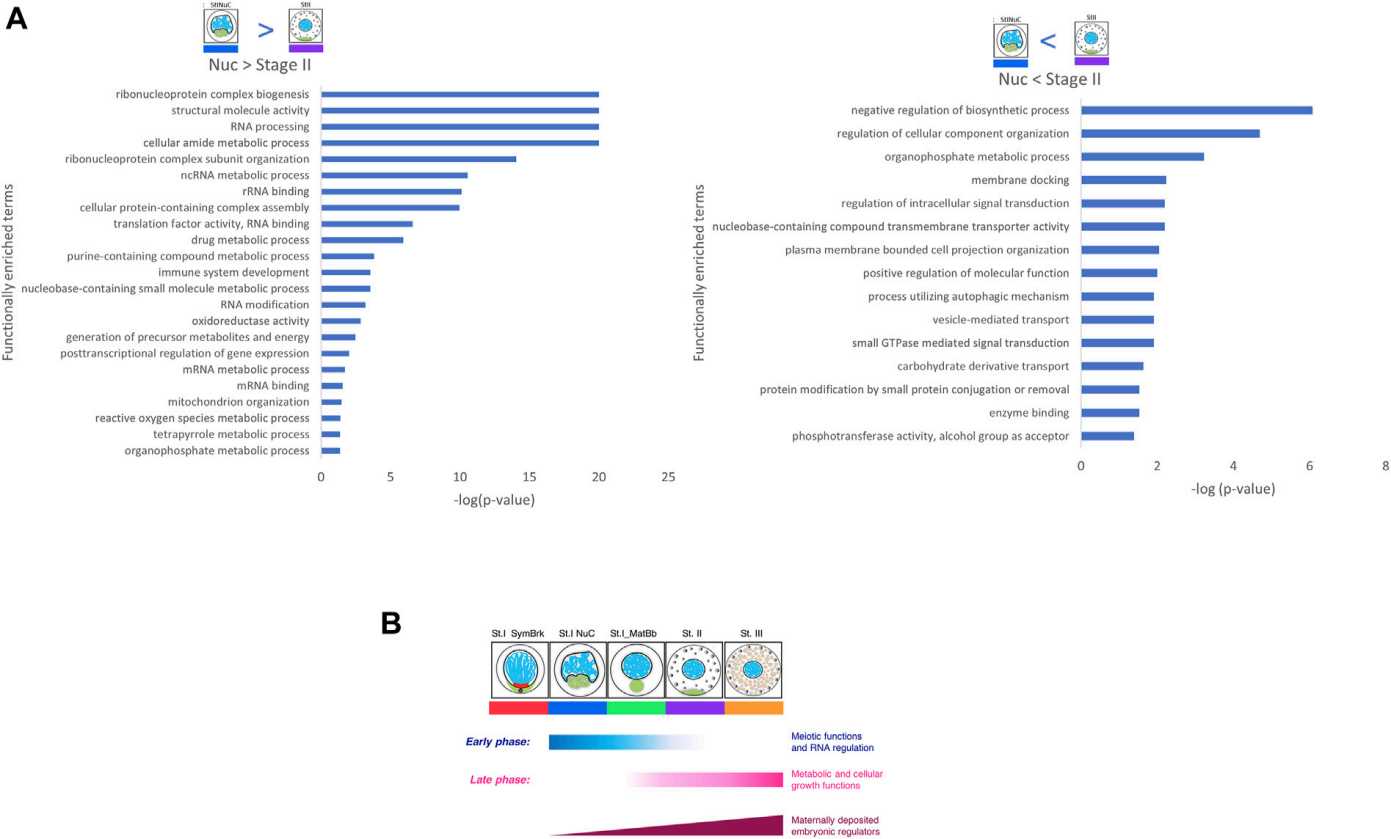
Functional analysis of pairwise comparisons. **(A)** Genes that were upregulated in Nuc over Stage II show many RNA functions, and genes that were up regulated in Stage II over Nuc show many biosynthesis, metabolic and membrane processes. **(B)** A scheme showing two phases of prominent differentiation activities in oogenesis determined by functional analysis of clusters and pairwise DEGs. The early phase includes mostly meiotic functions and RNA processing and regulation. The late phase includes mostly metabolic and cellular growth functions. Maternal embryonic regulators gradually increase their expression in parallel to these two phases.

Functional expression analysis revealed that DEGs that had higher expression in the early Nuc stage were associated with many functions related to RNA processing and DEGs with higher expression in the later Stage II were associated with vesicle and membranal functions (Figure 4A and similar results with other pairwise DEGs in Supplementary Figure S5). This is consistent with the two differentiation phases observed in our clustering analysis and discussed above.

## Discussion

Molecular mechanistic understanding of oogenesis is critical for advancing our knowledge of fertility, embryonic development, and female reproduction. Oogenesis has been described by ultrastructure, confocal, biochemical and molecular analyses (Elkouby et al., 2016; Elkouby and Mullins 2017a) and many key regulators have been identified by powerful forward and reverse genetics (Dosch et al., 2004; Wagner et al., 2004; Abrams et al., 2020). However, a comprehensive transcriptomic description of oocyte development in zebrafish has been lacking. Our work provides the first transcriptomic description of distinct stages throughout early oogenesis in zebrafish.

Transcriptome analyses of developing oocytes have been performed in *Drosophila* (Zhao et al., 2020a), fish (such as (Bobe et al., 2006; Luckenbach et al., 2008; Reading et al., 2012; Jamieson-Lucy et al., 2022)), mice (i.e (Nef et al., 2005; Zhao et al., 2018; Zhao et al., 2020b; Ge et al., 2021)), and mammals (reviewed in (Peng and Qiao 2021)). Many of these studies focused on specific stages of development, such as sex determination or late oogenesis. A recent study performed single cell RNA sequencing across most stages of mouse oocyte development (Niu and Spradling 2020). Although the research focused on follicle cell development, it is an important resource for the genetic changes that occur during oocyte development. A number of studies investigated the distinct transcriptomic changes in various stages during zebrafish oocyte development (for example (Wong et al., 2018; Zhu et al., 2018; Hong et al., 2019; Can et al., 2020; Cabrera-Quio et al., 2021)). Many of these focused on very specific oocyte stages or compared mutant and wild-type transcriptomes. However, to our knowledge, a comprehensive transcriptomic analysis of all specific stages in oogenesis has not been previously described in zebrafish.

In addition to the two rounds of whole genome duplications that occurred at the root of the vertebrate lineage, teleost fish experienced a third round of duplication. After duplication, the most likely fate of duplicated genes is the loss of one of the duplicates (Pasquier et al., 2016). Nonetheless, many genes in teleosts still have duplicates. These duplicates at times act in coordination (Zhou et al., 2008; Boyle-Anderson et al., 2022), and at times have specialized roles and expression (Yan et al., 2005; Kikuchi et al., 2020). In our dataset there were a large number of duplicated genes, and we chose to analyze only those genes whose expression was in the same direction (see Methods). It would be interesting to analyze the specific expression of duplicated genes and see if there is a difference in the effect on oogenesis between genes with the same directionality and genes with diverging expression.

There are many essential genes and known markers for oocyte development present in our datasets. First, key genes exhibit expected expression patterns and serve as controls for our analyses. First, pan germ cell markers such as piwi, dazl, and ddx4 (*vasa*) are present throughout oocyte development. Other markers like sycp1, a known meiotic marker transiently expressed in prophase (Gautier et al., 2013; Blokhina et al., 2019), is highly expressed specifically in the symmetry break stage in our dataset. Moreover, our analysis uncovered expression patterns of several genes that are concomitant with and likely underly their specific functions, like zar1, gdf9, and bmp15. Zar1 mutant oocytes do not progress from Stage Ib to Stage II and fish develop exclusively as males (Miao et al., 2016). In our dataset, zar1 is expressed in cluster #2 which contains genes highly expressed in early oogenesis stages, and less expressed in the transition to Stage II.

bmp15 and gdf9 are expressed mainly in oocytes. In contrast to mice, where gdf9 mutant oocytes are arrested, in zebrafish there was no detectable phenotype in gdf9 mutants. However, bmp15 mutants are arrested in Stage II with similar phenotypes to gdf9 mutants in mice (Dranow et al., 2016). In our dataset we do not see differential expression of gdf9, but bmp15 is expressed in cluster #3 with higher expression from the beginning of Stage II. These examples demonstrate that our analysis provides a reliable description of oogenesis, and therefore can predict previously unidentified processes and mechanisms.

Our functional enrichment analysis is of great interest and has been instrumental in providing new biological insights. First, somatic ovarian cells unexpectedly captured in the SymBrk group revealed many genes related to the immune system. The detection of many immune cells in this cell size is not surprising. However, potential roles for immune cells in zebrafish oogenesis and/or ovarian development have not been addressed and this would be very interesting to pursue in future investigation.

Second, functional terms that are specific to each cluster reveal previously uncharacterized stage appropriate processes. For example, glycosylation of protein is identified in cluster #3, which contains genes upregulated in Stage II and Stage III. Specifically, the process of N-glycosylation of protein is upregulated. In mice, gfp9 and bmp15 are N-glycosylated by dpagt1 (Li et al., 2020), which is upregulated in our dataset in Stage II and III compared to Nuc, reinforcing this prediction. Interestingly, the role of glycosylation in zebrafish oocyte development has not been fully investigated.

Furthermore, our pairwise comparisons revealed many DEGs between the stages we investigated, identifying genes that are specifically up- or down-regulated between stages. These genes could be used as markers to distinguish between stages and/or represent interesting candidates for functional studies. For example, CD82 is upregulated in the Nuc stage compared to the MatBb stage. Cd82 is a member of the tetraspanin family and has a known role in metastasis suppression (Yan et al., 2021). Such a role may be interesting in light of the major cellular reorganization that occurs during these stages, when oocytes transition from the germline cyst to the primordial follicle organization, or when oocyte-granulosa interactions reinforce as the follicle grows. However, Cd82 function has not been addressed in oocyte development in zebrafish.

Importantly, our analyses identified two developmental phases in oogenesis, with distinct prominent differentiation activities, accompanied by a major shift in gene expression between them (Figure 4B): 1) an early phase prominently executing meiotic functions and RNA processing and regulation, and 2) a late phase prominently executing metabolic and growth functions. Interestingly, we found that maternal embryonic regulators begin to accumulate early, and gradually increase their expression in parallel to these two phases (Figure 4B).

In summary, we provide a large dataset of stage specific oocyte gene expression that molecularly describes zebrafish oogenesis. This dataset can be further used for identifying additional processes and regulators, as well as more detailed information and markers (such as data from our 12 clusters for example) as first steps towards hypothesis driven functional studies. We hope this resource will be of use for continuing the efforts to uncover many yet unknown mechanisms underlying early oogenesis.

## Data Availability

The data presented in the study are deposited in the Sequence Read Archive repository, accession number SRP360207 and SRP360172.
