# Supplementary figures and images for: Stage Specific Transcriptomic Analysis and Database for Zebrafish Oogenesis
Source: Front Cell Dev Biol. 2022 Jun 6;10:826892. doi: 10.3389/fcell.2022.826892 (PMC9207522; doi:10.3389/fcell.2022.826892)

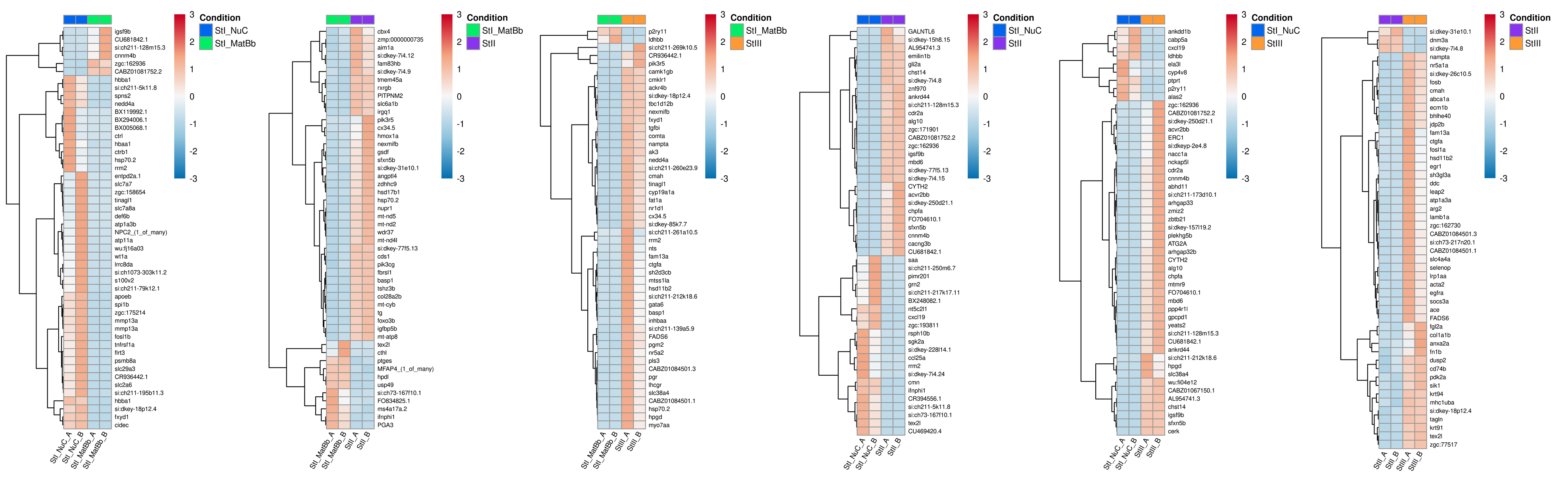

Supplement: Supplementary file 2 [file Image3.TIF]

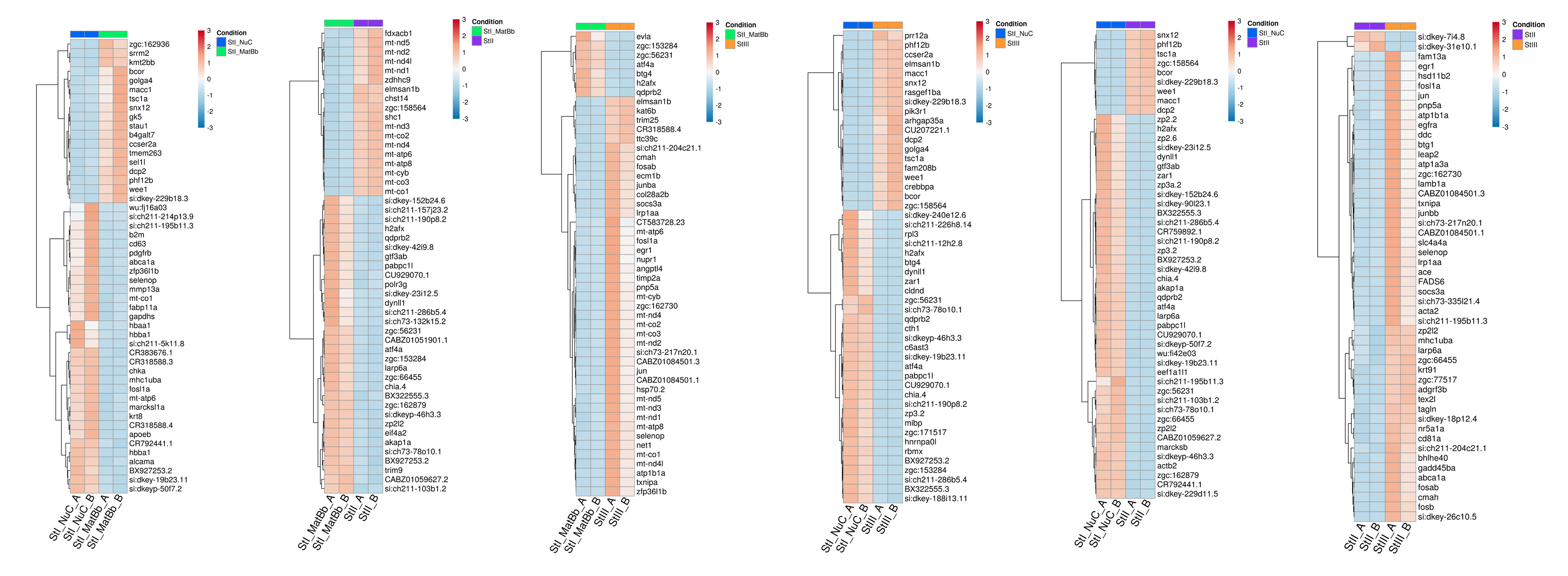

Supplement: Supplementary file 3 [file Image4.TIF]

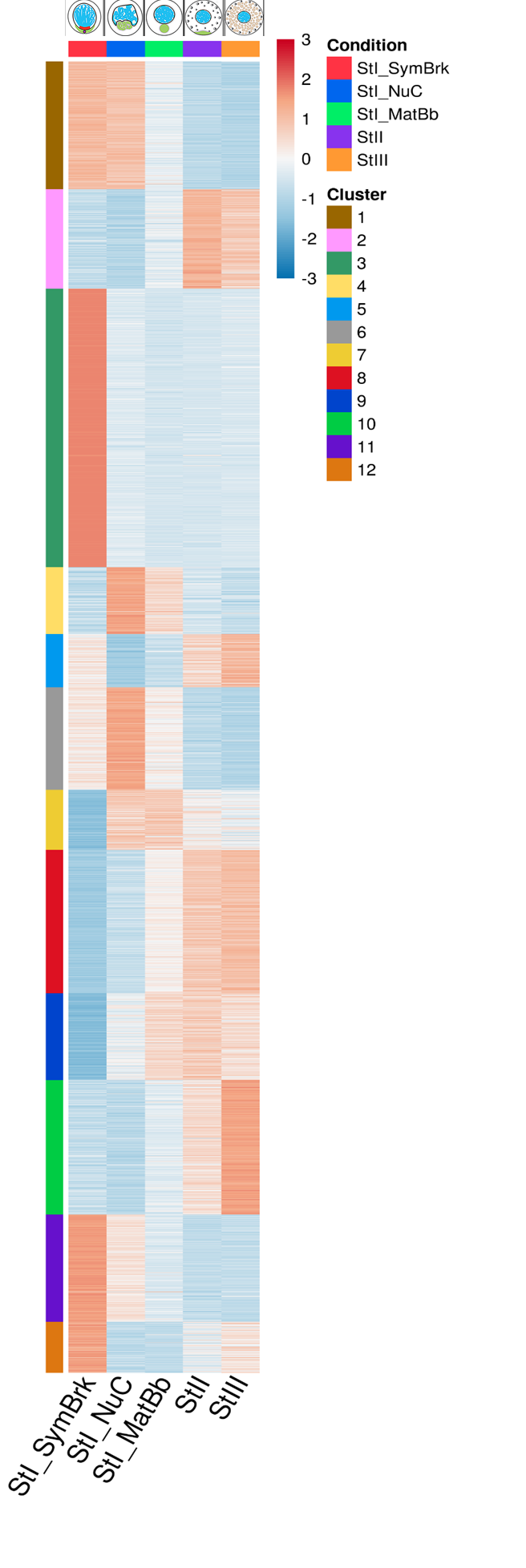

Supplement: Supplementary file 4 [file Image2.TIF]

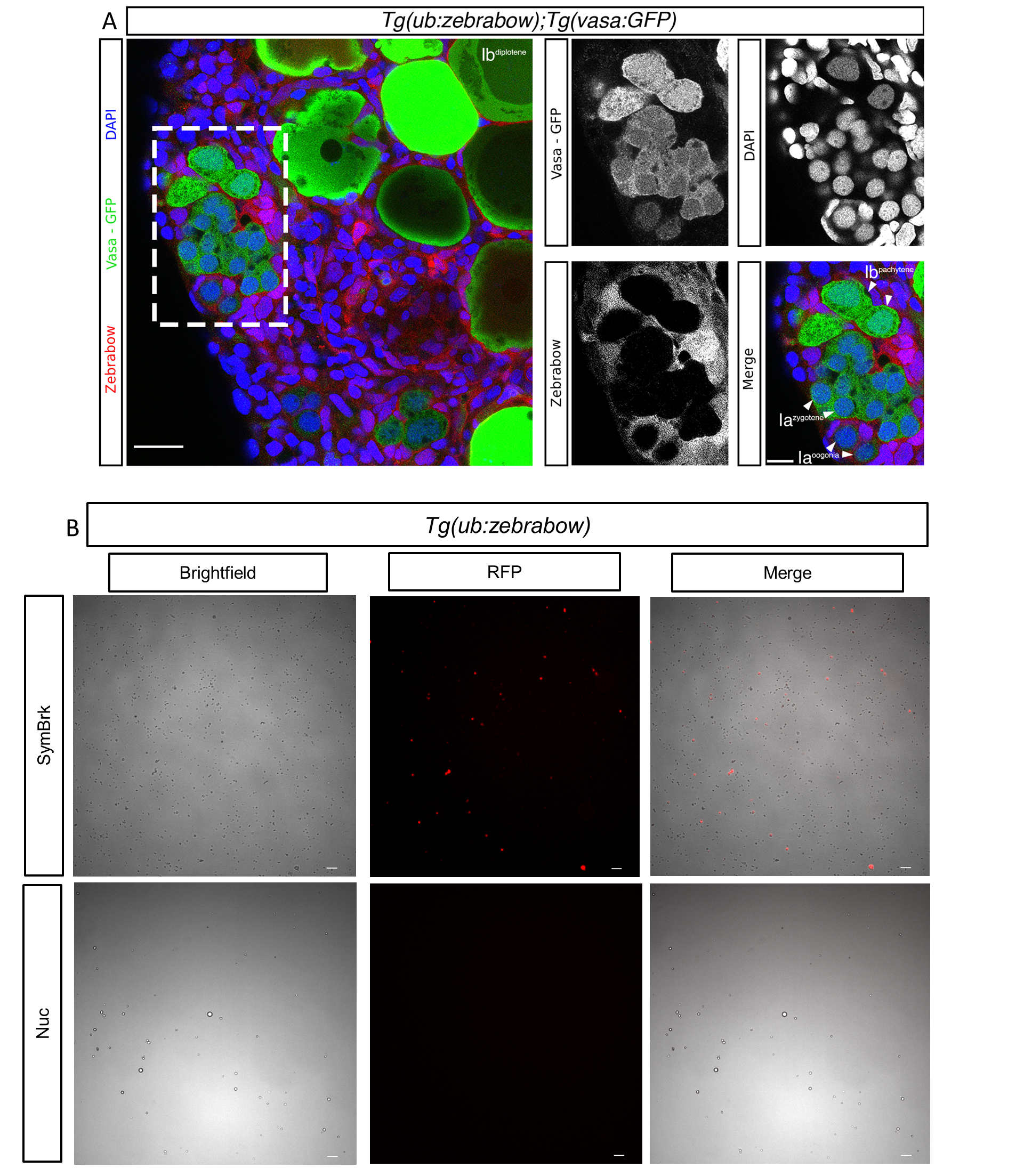

Supplement: Supplementary file 5 [file Image1.TIF]

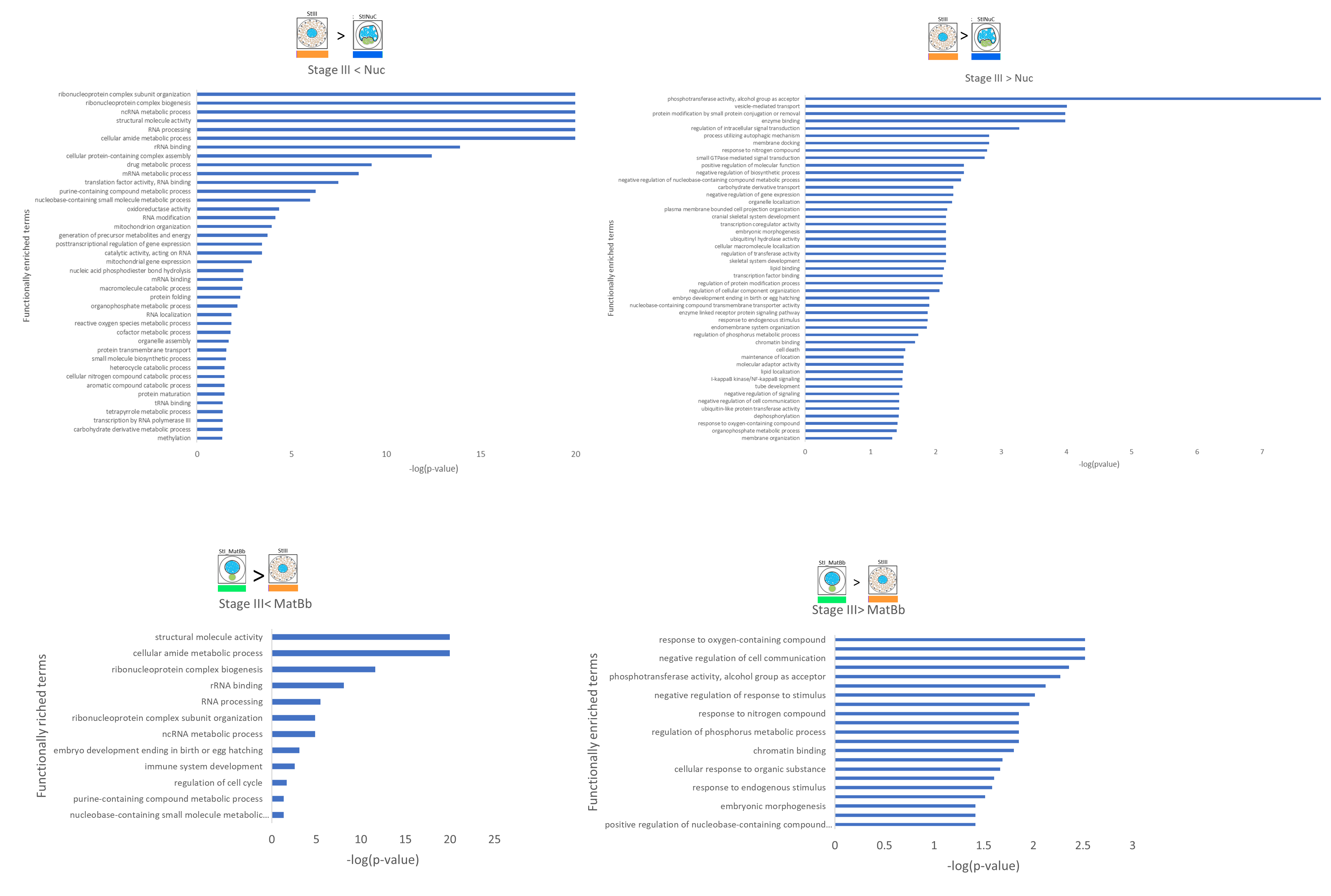

Supplement: Supplementary file 7 [file Image5.TIF]
